# Operative Management of Neonatal Lymphatic Malformations: Lesson Learned From 57 Consecutive Cases

**DOI:** 10.3389/fped.2021.709223

**Published:** 2021-08-18

**Authors:** Marianna Scuglia, Andrea Conforti, Laura Valfrè, Giorgia Totonelli, Chiara Iacusso, Barbara D. Iacobelli, Duino Meucci, Milena Viggiano, Fabio Fusaro, Andrea Diociaiuti, Francesco Morini, May El Hachem, Pietro Bagolan

**Affiliations:** ^1^Neonatal Surgery Unit, Medical and Surgical Department of the Fetus, Newborn, and Infant, Bambino Gesù Children's Hospital, IRCCS, Rome, Italy; ^2^Congenital Esophageal Disorders Unit, Medical and Surgical Department of the Fetus, Newborn, and Infant, Bambino Gesù Children's Hospital, IRCCS, Rome, Italy; ^3^Airway Surgery Unit, Department of Pediatric Surgery, Bambino Gesù Children's Hospital, IRCCS, Rome, Italy; ^4^Fetal Medicine and Surgery Unit, Medical and Surgical Department of the Fetus, Newborn, and Infant, Bambino Gesù Children's Hospital, IRCCS, Rome, Italy; ^5^Dermatology Unit and Genodermatosis Unit, Genetics and Rare Diseases Research Division, Bambino Gesù Children's Hospital, IRCCS, Rome, Italy; ^6^Department of Systems Medicine, Tor Vergata University, Rome, Italy

**Keywords:** lymphangioma, respiratory distress, neonates, sclerotherapy, OK432 sclerotherapy, EXIT procedure, multidisciplinary approach, lymphatic malformation

## Abstract

**Aim of the study:** Lymphatic malformations (LMs) are rare entities, sometimes difficult to treat, that may be life-threatening when intricately connected to airway structures. Invasive treatments are occasionally required, with sclerotherapy considered the treatment of choice and surgery as a second-line approach. The aim of the present study was to evaluate our multidisciplinary team experience in treating newborns affected by LMs requiring operative management, while defining early outcomes.

**Methods:** Retrospective review of all consecutive patients admitted for LMs requiring operative management between January 2000 and January 2019. Patients were mainly characterized based on anatomical district of the LM (and further stratified based on the development of respiratory distress), need for tracheostomy, number of sclerotherapies, indication for surgery, and residual disease beyond the 1st year. Morbidity and mortality were also evaluated. Fisher exact test and Mann–Whitney test were used as appropriate. Statistical significance was set at *p* < 0.05.

**Results:** Fifty-seven patients were included in the study, 36 with cervicofacial and/or mediastinal LMs and 21 with LMs of other anatomical districts. Due to the risk of developing respiratory distress at birth, patients with cervicofacial and/or mediastinal LMs were divided into two groups (8/36 group A vs. 28/36 group B). Group A patients are at higher risk for tracheostomy (7/8 group A vs. 1/28 group B, *p* = 0.0001) and more often require surgical reduction of the residual lymphatic abnormality (5/8 group A vs. 4/28 group B, *p* = 0.013). They also require sclerotherapies more often, but the difference is not statistically significant (8/8 group A vs. 19/28 group B, *p* = 0.15). Patients with cervicofacial/mediastinal LMs frequently suffer from persistent residual disease beyond the 1st year of life, significantly more often in group A (7/8 group A vs. 12/28 group B, *p* = 0.043).

**Conclusion:** LMs are rare conditions with potential life-threatening behavior. Their intrinsic clinical complexity requires a multidisciplinary approach to the affected patients. Planning a long-term follow-up is essential because of the late-term problems those patients may experience.

## Introduction

Lymphatic malformations (LMs) are congenital abnormalities of the lymphatic system composed of varying-sized lymphatic spaces and channels. More than half of all lesions develop on the head and neck (1). Although the exact pathogenesis remains uncertain, they are thought to arise from embryologic development disorders of the lymphatic system. It is thought that abnormal or absent communications between central venous sacs and the peripheral lymphatic system give rise to lymphatic accumulation and cyst formation (2).

Prenatal diagnosis of LM is reported, with lesions confirmed at birth in 50% of patients and usually observed as an asymptomatic mass (2, 3). Prenatal diagnosis of LM has significant clinical implications, because of the possible association with syndromes (e.g., chromosomal disorders such as Turner Syndrome or others). In addition, based on the anatomical location, LMs can be responsible for post-natal airway obstruction. Hence, prenatal diagnosis might influence prenatal pathway, modality, timing, and place of delivery. Specifically, prenatal detection of large cervicofacial, mediastinal, or thoracic LMs can suggest the possible need for emergent perinatal interventions to secure airways at the time of delivery. Consequently, these patients may require the presence of a skilled multidisciplinary team, experienced in *ex-utero* intrapartum treatment (EXIT).

LMs have traditionally been treated by surgical excision. Sclerotherapy has become increasingly popular during the past decade, becoming the primary treatment in macrocystic LMs (4). The use of OK-432 and other sclerosant agents (Fibrovein, bleomycin, ethanol, etc.) has been reported in the pediatric population, with good results and safety (2, 4–6).

**Figure 1:**
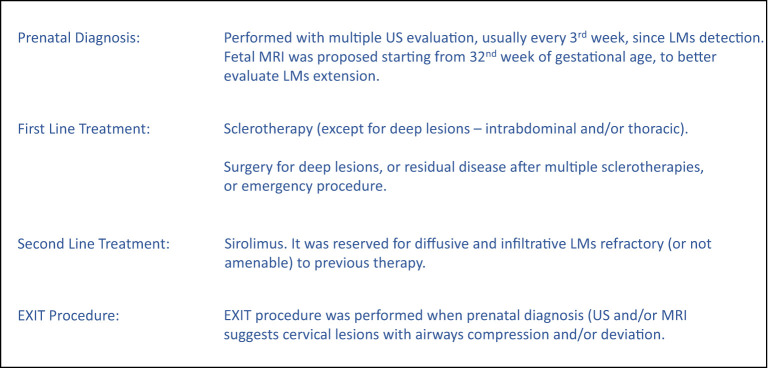
Treatment protocol.

The aim of the present study was to critically review the experience of our multidisciplinary team in the management of newborns with congenital LMs using a defined perinatal treatment protocol (Figure 1), to highlight risk factors for perinatal life-threatening events, while reporting early outcomes, within 1 year of life.

## Patients and Methods

All consecutive neonates with LMs, referred for possible operative management needs, between January 2000 and January 2019 were retrospectively reviewed. The Institutional Review Board approved the study waiving the need for informed consent because of its observational nature.

All cases were discussed and managed by a multidisciplinary team of pediatric surgeons, gynecologists, otorhinolaryngologists, interventional radiologists, neonatologists, dermatologists, anesthesiologists, ECMO team, and clinical geneticists.

Prenatal diagnosis was made *via* a combination of fetal ultrasound (US) and magnetic resonance imaging (MRI). Fetal MRI, when available, was usually performed during the third trimester of gestation, between the 29th and 32nd week.

Post-natal diagnostic confirmation was based on clinical examination, US, and MRI. Cytology or histology confirmed the diagnosis in patients undergoing sclerotherapy or surgery, respectively.

LMs were classified according to ISSVA classification: (1) macrocystic LM, with more than 50% of the cysts larger than 1 cm in diameter; (2) mixed LM, with <50% of the cysts larger than 1 cm in diameter; and (3) microcystic LM, with all cysts <1 cm (7).

LMs are mainly located on the head and neck. Patients with cervicofacial and/or mediastinal LMs may develop respiratory distress soon after birth due to the presence of LMs strictly linked to airway structures. For this reason, these patients were divided into two groups (group A and group B) based on the presence/absence of acute severe respiratory distress within 6 h after birth to evaluate possible risk factors specifically related to developing such symptoms (Figure 1).

Patients' demographics, disease characteristics including anatomical sites and lesion size, rate of prenatal diagnosis, termination of pregnancy (ToP), number of sclerotherapies during the 1st year, sclerotherapy-related complications, need for surgery, need for tracheostomy, and presence of residual disease within the 1st year were recorded. Morbidity and mortality were also evaluated.

### Delivery

In case of LM prenatal diagnosis, delivery was planned *via* cesarian section when LMs were judged to potentially cause perinatal problems, with possible requirement of neonatal resuscitation and early post-natal treatment. Specifically, cervical LMs may cause airway obstructions exposing fetuses to difficult airway management and stabilization. Those cases were collegially discussed and eventually the *ex utero* intrapartum treatment (EXIT) procedure was proposed:

EXIT was performed when cervical LMs determine anatomic compression and/or deviation of the trachea, having a diameter higher than 5 cm at prenatal US/MRI.

### Sclerotherapy

In the last 20 years, we have been using sclerotherapy as the initial treatment choice for LMs. Indication for sclerotherapy was given by the multidisciplinary team based on patient age, clinical characteristics of LMs, and the anatomical sites involved. Deep lesions (intra-abdominal/thoracic) were excluded from sclerotherapy for intrinsic difficulties of puncture and for the risk of post-sclerotherapy complications such as intestinal occlusion or thoracic compressive side effects.

Cytological analysis and lymphocyte rate >75% confirmed LM diagnosis. In addition, intraoperative fluoroscopy with injection of hydrosoluble contrast medium was performed to confirm cyst communication and exclude abnormal vascular shunt. OK-432 sclerotherapy was performed under sedation/general anesthesia, and doses were adjusted according to Ogita et al. (4). Mild fever, local heating, and skin erythema were considered as a positive inflammatory reaction to the therapy in most cases. No steroids or antibiotic therapy was started as a rule when these clinical signs were present.

### Surgery

Surgical approach was reserved for selected patients with residual disease after multiple sclerotherapies (at least three administrations) with no residual cysts, as an emergency procedure for intractable respiratory distress secondary to mass compression, or as an elective procedure in cases of intra-abdominal and thoracic lesions. Surgical treatment was ideally aimed to reach complete and safe disease removal. Nonetheless, incomplete resection was always considered when radical excision seemed too risky for iatrogenic injuries (neurological and vascular) and functional or cosmetic impairment.

In case of tongue involvement with severe protrusion, causing respiratory and/or long-term swallowing and feeding difficulties, reduction surgery (usually after 1 year of age) was considered, generally after tracheostomy.

### Sirolimus

Systemic pharmacological treatment with sirolimus was in the last years considered for patients with diffuse and infiltrative LMs refractory to previous therapy, and/or when surgical approach is not feasible due to the risk of permanent iatrogenic damage (e.g., blindness).

When used, the treatment was initially administrated in a hospital environment for monitoring possible development of adverse events. The dosage was 0.4–0.8 mg/m^2^ twice daily to maintain sirolimus-blood levels in range (<10 ng/ml) (8–10). Subsequently, clinical, pharmacological, and radiological follow-up was started.

### Statistical Analysis

Statistical analysis was performed using Fisher exact test and Mann–Whitney test as appropriate. *p* < 0.05 was considered significant. Data are presented as prevalence or medians and interquartile ranges (IQRs).

## Results

**Figure 2:**
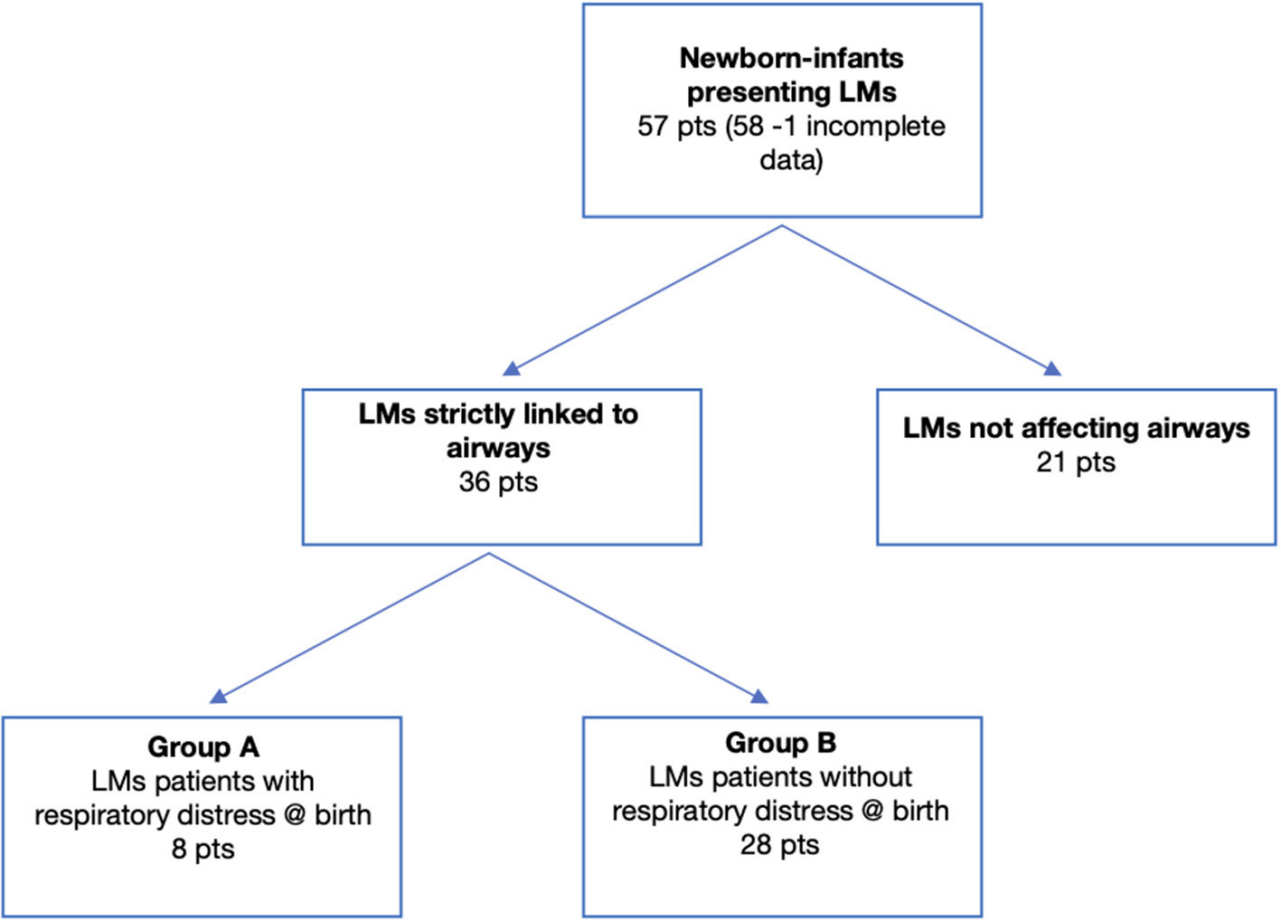
Consort diagram.

During the study period, 58 patients with LMs required an operative management. One patient was excluded due to poor data, leaving 57 patients available for the study. Thirty-six patients presented with cervicofacial and/or mediastinal LMs linked to airway structures (63%), while LMs did not involve the airways in 21 patients (37%). Patients affected by cervicofacial and/or mediastinal LMs were further subdivided into two subgroups, based on the presence (8 patients, group A) or absence (28 patients, group B) of acute severe respiratory symptoms at birth or within 6 h after birth, due to mediastinal shifting and/or airway compression (Figure 2).

No significant differences were found on US appearance of LMs. Macrocystic lesions were the most represented in our population (77%), and 39 patients (68%) presented major lesions (>3 cm diameter).

Three out of 41 patients with prenatal diagnosis and a cervical LM > 5 cm in diameter were considered at high risk of airway obstruction at birth and therefore managed with an EXIT procedure (7%). The EXIT procedure allowed safely intubating these patients.

Overall, 22 patients were exclusively treated with sclerotherapy (38%) with one or more OK-432 applications. Surgery alone was performed in 15 patients (26%), while in 13 infants, surgery was required after sclerotherapy (23%). Seven infants with small and/or asymptomatic LMs were managed conservatively with close monitoring (12%), and a spontaneous regression of the lesions was observed. Two infants were additionally treated with sirolimus according to the neonatal protocol (3%).

Twenty-three patients out of 51 (45%) presented with residual disease at 1 year of age.

One patient died at 3 months of age due to complications of metapneumovirus pneumonia. This patient was treated with sclerotherapy and subsequently with sirolimus (8).

Overall, 51 patients reached a minimum of 1-year follow-up. Table 1 summarizes the main findings.

**Table 1:** Overall demographics, clinical characteristics, and treatment options.

		**Group A**	**Group B**	**p**
		**8 pts**	**28 pts**	
Birth weight, gr; median (IQR)		3,030 (2947.5-3,175)	3,185 (3,030–3,523)	0.28
Gestational age, weeks; median (IQR)		38 (37–38)	39 (38.25–40)	0.053
Prenatal diagnosis, n (%)		7 (87)	18 (64)	1
Exit, n/n prenatally diagnosed (%)		2/7 (28)	1/18 (5.5)	0.11
Site, n (%)	Oral cavity	8 (50)	3 (11)	**0.0001**
	Neck	8 (100)	23 (82)	0.56
	Mediastinum/thorax	2 (25)	6 (21)	1.0
Macrocystic disease, n (%)		8 (100)	23 (82)	0.56
Microcystic disease, n (%)		–	–	1.0
Mixed disease, n (%)		–	5 (18)	0.56
Overall size > 3 cm, n (%)		8 (100)	19 (68)	0.15
Respiratory distress, n (%)		7 (87)	1 (4)	**0.0001**
Tracheostomy, n (%)		7 (87)	1 (4)	**0.0001**
Sclerotherapy, n (%)		8 (100)	19 (68)	0.15
N° sclerotherapy, median (IQR)		2.5 (1.75–3.25)	1 (1, 2)	0.21
Surgery, n (%)		5 (62.5)	8 (29)	0.10
Residual disease at 1 year, n (%)		7 (87.5)	12 (43)	**0.043**
Residual disease (sclerotherapy), n (%)		3 (37.5)	8 (29)	0.67
Residual disease (surgery), n (%)		–	–	1
Residual disease (sclerotherapy + surgery), n (%)		4 (50)	4 (14)	0.053
Sirolimus, n (%)		1 (12.5)	1 (3.5)	0.4
Deaths, n (%)		–	1 (3)	1.0

*Bold values highlights statistical significance*.

Group A vs. Group B (Table 2).

**Table 2:** Comparison on demographics, clinical characteristics, and treatment options between patients with or without respiratory distress (group A and group B, respectively).

	**Overall 57 pts**
Birth weight, gr; median (IQR)	3,160 (3,025–3,465)
Gestational age, weeks; median (IQR)	39 (37–39.5)
Prenatal diagnosis, n (%)	41 (72)
Exit, n/n prenatally diagnosed (%)	3/41 (7)
LMs cervico-Mediastinal/linked to airways structures, n (%)	36 (63)
Head and Neck, n (%)	34 (60)
Thorax, n (%)	13 (23)
LMs not affecting airways, n (%)	21 (37)
Abdomen, n (%)	12 (18)
Other sites, n (%)	8 (14)
Macrocystic disease, n (%)	44 (77)
Microcystic disease, n (%)	2 (3.5)
Mixed disease, n (%)	9 (16)
Overall size > 3 cm, n (%)	39 (68)
Respiratory distress, n (%)	8 (14)
Tracheostomy, n (%)	8 (14)
Sclerotherapy, n (%)	35 (61)
N° sclerotherapy, median (IQR)	1 (1–3)
Surgery, n (%)	28 (49)
Residual disease at 1 year, n (%)	23 (40)
Residual disease (sclerotherapy), n (%)	12 (21)
Residual disease (surgery), n (%)	–
Residual disease (sclerotherapy + surgery), n (%)	11 (19)
Sirolimus, n (%)	2 (3.5)
Deaths, n (%)	1 (1.7)

Eight patients had symptoms within the first 6 h of life (group A), while 28 did not (group B). The two groups had similar birth weight and gestational age. The vast majority (69%) had prenatal diagnosis with no difference between the two groups.

Largely, neck swelling was the most common LM clinical presentation in both A and B patients (8/8 patients, 100% vs. 23/28 infants, 82%; *p* = 0.56). Interestingly, all patients who experienced respiratory distress at birth (group A) presented oral LMs extension. Conversely, only one infant in group B had LM arising from the oral cavity (*p* < 0.0001).

All but one group A patient required tracheostomy for persistent respiratory distress and possible difficult intubation, while the only patient in group B with oral cavity involvement required tracheostomy for progressive swelling of the tongue leading to increasing respiratory failure. Seeing these results differently, all except one patient with oral involvement required a tracheostomy vs. none of those without oral involvement (8/9 vs. 0/27; *p* < 0.0001). Based on prenatal findings, three EXIT procedures were performed, two in group A and one in group B.

No differences were observed also in terms of number of sclerotherapies [2.5 sclerotherapies (IQR 1.75–3.25) group A vs. 1 sclerotherapy (IQR 1–2) group B, *p* = 0.21] and need for surgical removal [5/8 patients (62.5%) group A1 vs. 8/28 patients (29%) group A2, *p* = 0.10].

Two patients, one per group, were further treated with sirolimus [1/8 patients (12.5%) group A vs. 1/28 patients (3.5%) group B, *p* = 0.4] because they were unresponsive to sclerotherapy and with high risk of post-operative disabilities.

The persistence of residual disease beyond 1 year of age was significantly higher in group A in comparison with group B patients (87.5 vs. 43%, *p* = 0.043). No emergency surgery was needed, since all patients who presented respiratory distress were primarily intubated and eventual tracheostomy was performed. Table 2 summarizes the main results.

## Discussion

**Figure 3:**
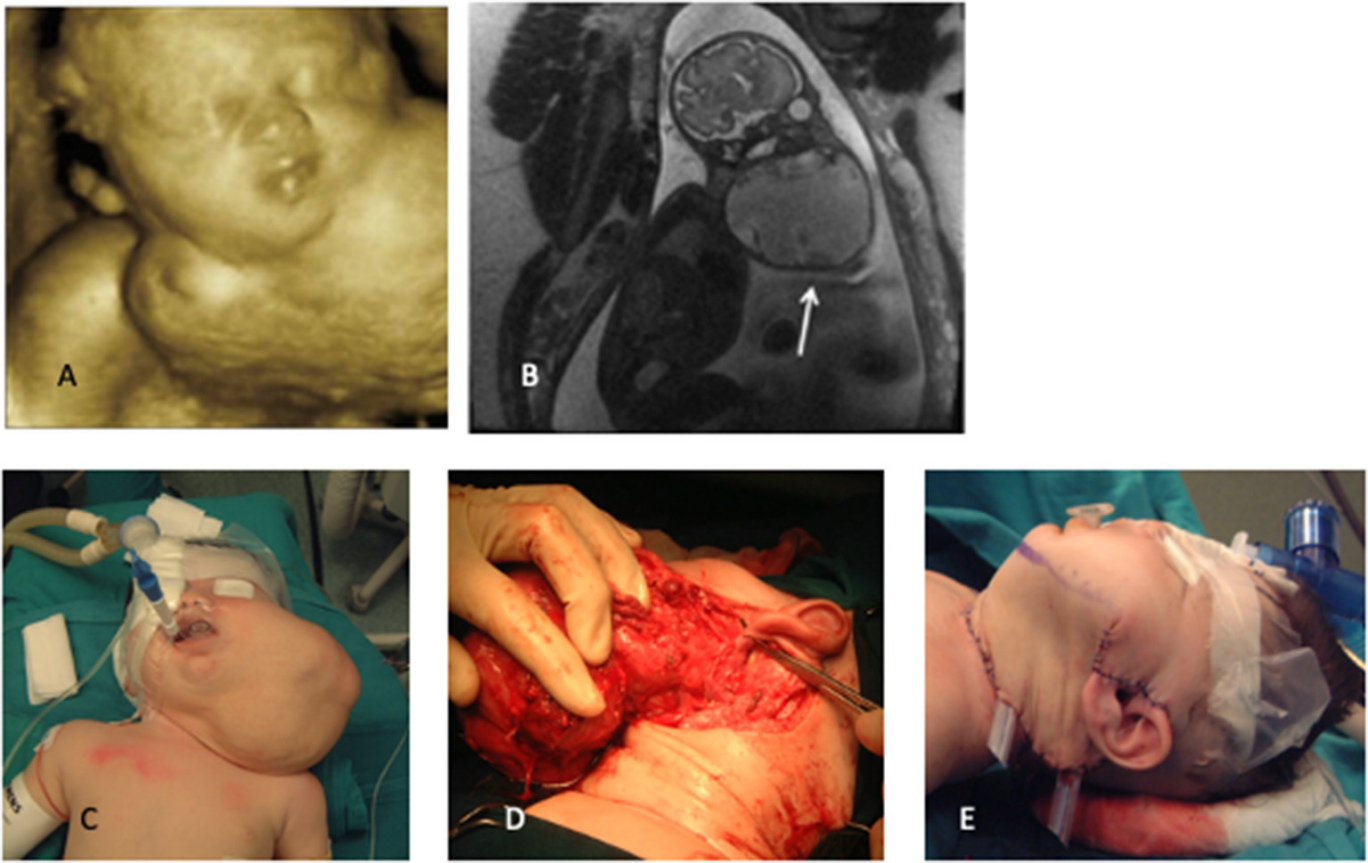
**(A)** 3D prenatalxc ultrasound, **(B)** fetal MRI, **(C)** preoperative appearance, **(D)** intraoperative dissection, **(E)** final reconstruction. White arrow indicate LM.

**Figure 4:**
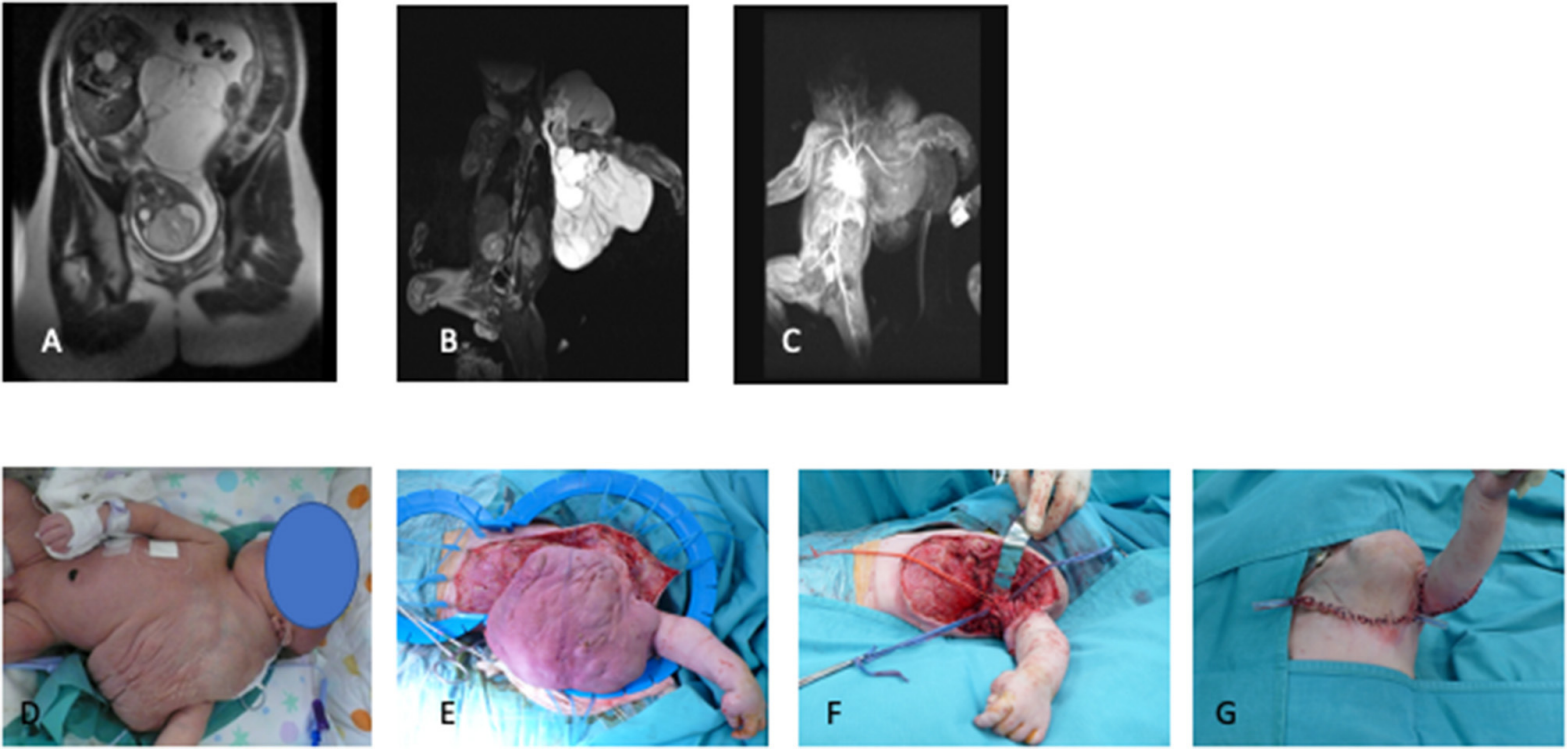
**(A)** Fetal MRI, **(B,C)** postnatal T2-weighted MRI sequences, **(D)** preoperative appearance, **(E,F)** intraoperative dissection, **(G)** final surgical reconstruction.

**Figure 5:**
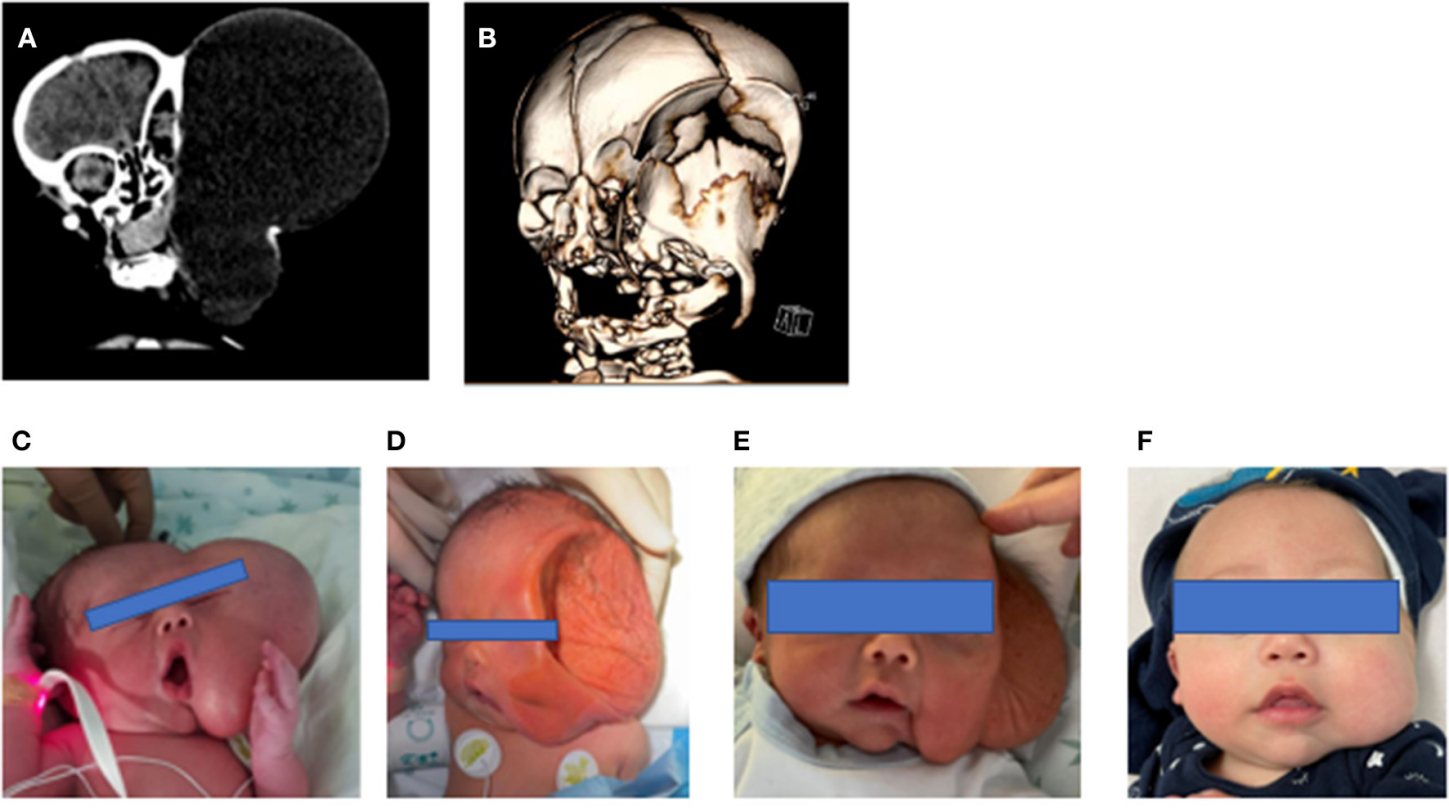
**(A)** Postnatal TC scan, **(B)** 3D CT reconstruction, **(C)** pre-sclerotherapy appearance, **(D–F)** post-sclerotherapy evolution.

LMs represent a wide spectrum of rare diseases (11), frequently diagnosed in the fetus, with involvement of head and neck. Wide spectrum in size, extent, and natural history of lymphatic anomalies have been reported, both prenatally and postnatally (2) (Figures 3–5). Therefore, treatment pathways should be individualized based on clinical features and radiological appearance. To our knowledge, this is the largest series reporting on perinatal treatment of LMs in newborn babies.

Prenatal US diagnosis of cervical LMs was firstly described 40 years ago (12). Long-term prognosis of these prenatally detected lesions is poorly reported; they were significantly coupled with genetic abnormalities (13, 14) ultimately associated with fetal demise (FD) or termination of pregnancy (ToP). In the early 2000, Fujita and coworkers described a high prevalence of FD and/or ToP or early neonatal deaths for perinatally diagnosed LMs (31/36 cases, 86%) (14). In our series, prenatal detection rate was 72% (41/57 patients). Noteworthy, FD and/or ToP was not observed in our cohort of patients. A possible reason could probably be a selection bias due to the “late” (after 18 weeks) patients' presentation to our Prenatal Counseling Service (Italian legislation allows voluntary termination of pregnancy until completion of the 22nd week of gestational age). A second possible reason could be the high parental motivation to continue their pregnancies. Thirdly, it is possible that improved diagnostic skills also allow to detect prenatally less severe LMs, thereby ameliorating the overall outcome of prenatally diagnosed LMs.

**Figure 6:**
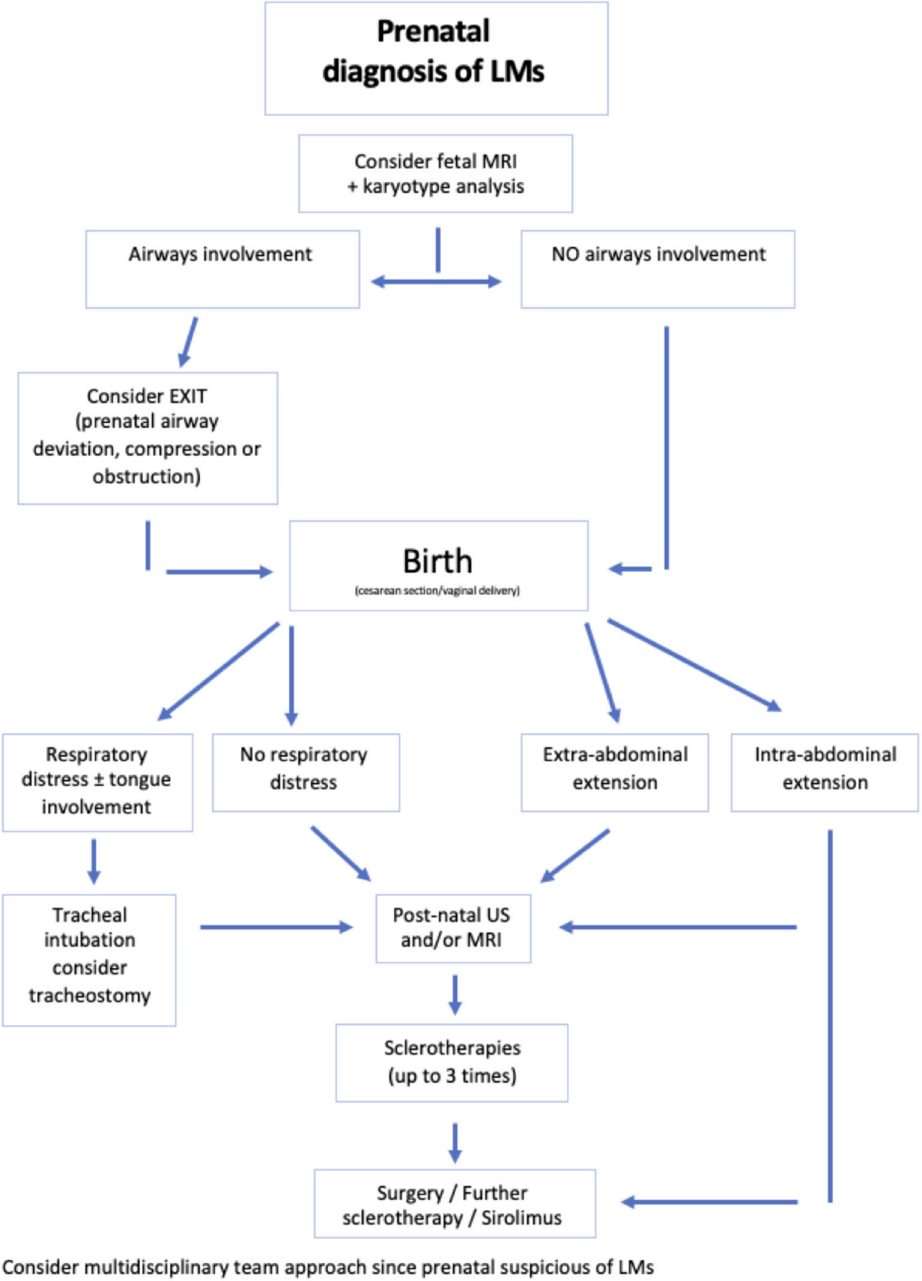
Proposed treatment algorithm.

Different anatomical locations of LMs led to diverse clinical manifestations and distinctive management pathways. Consistently with the available literature (15), our data suggest that patients with a LM not close to airways have low risk of life-threatening difficulties and they can be electively approached, either by surgery (e.g., abdominal LMs) or by sclerotherapy. Conversely, those infants with neck and thorax LMs often connected to the airways are at risk of respiratory distress at or soon after birth, requiring EXIT or emergency intensive care and/or surgery. In our series, newborns with respiratory distress at birth mainly had concomitant tongue involvement, while neck swelling was the most frequent presentation. Additionally, all except one patient with tongue involvement underwent a tracheostomy, vs. none of the patients with neck/mediastinal LMs without tongue involvement. This is consistent with the reported experience by Laie et al. who described 13 cases of EXIT in fetuses prenatally diagnosed with cervical LM (16). In our center, criteria to plan and perform EXIT were deviation, compression, or obstruction of the airways. Consistent with the literature, in our population, three EXIT procedures were performed after a multidisciplinary extensive discussion of all patients presenting LMs tightly linked to the airway structures. Interestingly, all patients with prenatal detection of tongue involvement (7/57, 12%) and no appearance of direct airway compression/obstruction were excluded from EXIT. However, they frequently experienced significant respiratory symptoms at birth or soon after and often required emergency intubation or tracheostomy (7/57 12%). Furthermore, the only patient who required tracheostomy without presenting respiratory symptoms at birth had LM affecting the floor of the mouth. EXIT is a complicated procedure with a high risk of perinatal mortality and adverse events, and it is also associated with maternal risk. Therefore, this procedure should be reserved for cases with fetal airway obstruction and only in a tertiary center with a rapid access to the neonatal intensive care, guaranteeing low maternal risk (17). Based on these findings, it is possible to conclude that patients prenatally diagnosed with tongue involvement have a high chance to manifest respiratory distress at birth and require tracheotomy soon thereafter. Therefore, oral cavity involvement should also be included in the criteria indicating EXIT procedure. Since a structured approach to LMs was seldom proposed (18), with treatment algorithms suggested only for those lesions in the head and neck (19, 20), based on our results, we propose a comprehensive LM management algorithm (Figure 6).

In patients with LMs, the decision between observation, sclerotherapy, and excision is complex. Currently, there is no evidence in the literature to define a standardized best choice for primary approach (21). Since its introduction in 1987 (4), sclerosing therapy with intra-cystic injection of OK-432 has progressively become the first-line treatment for LMs, with different reports concluding that OK-432 is safe and effective (22, 23). OK-432 has the ability to induce panendothelial destruction thanks to its immunomodulatory activity. Also, it does not possess any systemic toxicity due to its capacity to reach a maximal contact with the vascular endothelium while minimizing extravasation into the surrounding tissue (5, 24). The efficacy of OK-432 in treating LMs has been widely discussed: initial reports were extremely encouraging, describing almost complete resolution in all patients (4), while later papers were more cautious (18, 25). Moreover, surgery after OK432 is not difficult and allows one to obtain good cosmetic results. This sclerosant agent, when compared with other agents, seems to be the most effective for macrocystic lymphangiomas and macrocystic components of mixed lesions (23, 24). In our experience, OK432 sclerotherapy was safe and partially effective in newborn infants with LMs located close to the airways. In these patients, sclerotherapy allowed the reduction of the lesion, especially of the major cysts, usually following LM swelling due to the inflammation induced by therapeutic agents. Particular attention must be paid to the possible side effects of sclerotherapy, such as the possible ventilatory needs secondary to the inflammatory reaction (and the consequent distension of the treated lymphatic cysts) of sclerotherapy. Several other sclerosing agents have been used: doxycycline, sodium tetradecyl sulfate, dehydrated ethanol, betadine, polidocanol, sodium morrhuate, and bleomycin (26). Bleomycin and doxycycline are widely used for their effectiveness and safety. Bleomycin is an antitumoral antibiotic that causes minimal inflammation and swelling and is indicated for LMs around sensitive structure, while doxycycline is a tetracycline antibiotic that has therapeutic effects on LMs thanks to its antitumoral property to suppress vascular endothelial growth factors during lymphangiogenesis (26–28).

In more than half of our patients with cervicofacial/mediastinal LMs, sclerotherapy alone was able to cure the malformation. When it is incompletely effective, sclerotherapy allows postponing surgery and performing a less invasive excision. Surgery may be required for persistent or recurrent LMs, in order to improve functional status and esthetical outcome. In our series, 49% of patients with cervicofacial and/or mediastinal LMs required surgery for the malformation that persisted after sclerotherapy: it was mainly required in those with perinatal respiratory distress (62.5%, group A), and less needed for group B patients (29%). This was in keeping with the experience of other centers where surgical excision was required in almost half of the patients treated for head and neck LMs (2, 16, 19, 29).

Primary surgical excision of LMs was commonly reserved to cases with localized and confined abdominal/thoracic LM (28/57, 49%) in which surgery was thought to be safe with no risk for neuro-vascular sequelae. Specifically, abdominal LMs, usually originating in the mesenteric region, were surgically treated and completely excised. Indeed, this technique has been historically considered the treatment of choice for abdominal LMs (30). However, a high complication rate (bleeding, iatrogenic damages, and deformity) has also been described (31), leading some authors to propose sclerotherapy also for cases with abdominal LMs, reporting good results (30, 32, 33).

Sirolimus is an mTOR inhibitor mainly used as an immunosuppressive agent after organ transplantation. Sirolimus inhibits angiogenesis through the AKT/PIK3 pathway, and for this reason, in the last years, it has been proposed for the treatment of vascular anomalies, including LMs. Although sirolimus was initially administered for compassionate use, nowadays, it is a suitable therapy for diffuse and infiltrative LMs in children (34). According to Czechowicz et al., we use a neonatal dosing due to the reduced hepatic metabolism of sirolimus in neonates (9, 10).

The safety profile of sirolimus in the treatment of LMs appears favorable. However, a recent survey reported that patients with LMs treated with sirolimus could experience infectious complications and severe adverse events, including death (8). Therefore, the authors suggested to carefully monitor these young patients for toxicity and side effects (8). Also, the authors recommended to administer vaccinations before starting sirolimus treatment and continue to provide dead vaccines according to recommendations (8).

The persistence of residual LM was mainly observed in patients with cervicofacial and/or mediastinal LMs due to the involvement of the floor of the mouth or the tongue that makes radical surgical often impossible (53%). In these complex patients, treatment of residual disease (either with further sclerotherapy or surgery) after the 1st year of life should be personalized based on site and extension of the lesion, residual functional or severe cosmetic problems, and treating center experience.

Limitations of the present study include the relatively small number of eligible patients, its retrospective nature, and the lack of genetic testing, with only a very few cases treated with sirolimus.

## Conclusion

LMs, in particular those closely linked to the airways, still represent a challenging condition. Patients presenting LMs involving the tongue are at higher risk for respiratory distress at birth and frequently require tracheostomy. When prenatally detected, tongue involvement is an absolute indication to EXIT procedure and, possibly, to early tracheostomy. Therefore, a multidisciplinary team is of paramount importance in the perinatal management of patients with LMs. Although sclerotherapy remains the first-line treatment, a large number of patients require radical or debulking surgery to maximize functional and cosmetic outcomes. The reported experience and the proposed algorithm may be of help for a more focused counseling aiming to obtain the best possible detailed information.

## Data Availability Statement

The raw data supporting the conclusions of this article will be made available by the authors, without undue reservation.

## Ethics Statement

The studies involving human participants were reviewed and approved by Bambino Gesù Children's Hospital Ethical Committee. Written informed consent from the participants' legal guardian/next of kin was not required to participate in this study in accordance with the national legislation and the institutional requirements.

## Author Contributions

AC and MS have to be considered first co-author. AC, MS, ME, and PB contributed to conception and design of the study. LV, GT, and AD analyzed the data. LV, CI, and BI processed the figures. AC and MS wrote the first draft of the manuscript. DM, MV, FF, and FM wrote sections of the manuscript. All authors contributed to the article and approved the submitted version.

## Conflict of Interest

The authors declare that the research was conducted in the absence of any commercial or financial relationships that could be construed as a potential conflict of interest.

## Publisher's Note

All claims expressed in this article are solely those of the authors and do not necessarily represent those of their affiliated organizations, or those of the publisher, the editors and the reviewers. Any product that may be evaluated in this article, or claim that may be made by its manufacturer, is not guaranteed or endorsed by the publisher.
